# Promoter variations in DBR2-like affect artemisinin production in different chemotypes of *Artemisia annua*

**DOI:** 10.1093/hr/uhad164

**Published:** 2023-08-16

**Authors:** Xingwen Wang, Lan Wu, Li Xiang, Ranran Gao, Qinggang Yin, Mengyue Wang, Zhaoyu Liu, Liang Leng, Yanyan Su, Huihua Wan, Tingyu Ma, Shilin Chen, Yuhua Shi

**Affiliations:** Key Laboratory of Beijing for Identification and Safety Evaluation of Chinese Medicine, Artemisinin Research Center, Institute of Chinese Materia Medica, China Academy of Chinese Medical Sciences, Beijing 100700, China; Key Laboratory of Beijing for Identification and Safety Evaluation of Chinese Medicine, Artemisinin Research Center, Institute of Chinese Materia Medica, China Academy of Chinese Medical Sciences, Beijing 100700, China; Key Laboratory of Beijing for Identification and Safety Evaluation of Chinese Medicine, Artemisinin Research Center, Institute of Chinese Materia Medica, China Academy of Chinese Medical Sciences, Beijing 100700, China; Key Laboratory of Beijing for Identification and Safety Evaluation of Chinese Medicine, Artemisinin Research Center, Institute of Chinese Materia Medica, China Academy of Chinese Medical Sciences, Beijing 100700, China; Key Laboratory of Beijing for Identification and Safety Evaluation of Chinese Medicine, Artemisinin Research Center, Institute of Chinese Materia Medica, China Academy of Chinese Medical Sciences, Beijing 100700, China; Key Laboratory of Beijing for Identification and Safety Evaluation of Chinese Medicine, Artemisinin Research Center, Institute of Chinese Materia Medica, China Academy of Chinese Medical Sciences, Beijing 100700, China; Institute of Herbgenomics, Chengdu University of Traditional Chinese Medicine, Chengdu 611137, China; Institute of Herbgenomics, Chengdu University of Traditional Chinese Medicine, Chengdu 611137, China; Amway (China) Botanical R&D Center, Wuxi 214115, China; Key Laboratory of Beijing for Identification and Safety Evaluation of Chinese Medicine, Artemisinin Research Center, Institute of Chinese Materia Medica, China Academy of Chinese Medical Sciences, Beijing 100700, China; Key Lab of Chinese Medicine Resources Conservation, State Administration of Traditional Chinese Medicine of the People's Republic of China, Institute of Medicinal Plant Development, Chinese Academy of Medical Sciences & Peking Union Medical College, Beijing 100193, China; Key Laboratory of Beijing for Identification and Safety Evaluation of Chinese Medicine, Artemisinin Research Center, Institute of Chinese Materia Medica, China Academy of Chinese Medical Sciences, Beijing 100700, China; Institute of Herbgenomics, Chengdu University of Traditional Chinese Medicine, Chengdu 611137, China; Key Laboratory of Beijing for Identification and Safety Evaluation of Chinese Medicine, Artemisinin Research Center, Institute of Chinese Materia Medica, China Academy of Chinese Medical Sciences, Beijing 100700, China

## Abstract

*Artemisia annua* is the only known plant source of the potent antimalarial artemisinin, which occurs as the low- and high-artemisinin producing (LAP and HAP) chemotypes. Nevertheless, the different mechanisms of artemisinin producing between these two chemotypes were still not fully understood. Here, we performed a comprehensive analysis of genome resequencing, metabolome, and transcriptome data to systematically compare the difference in the LAP chemotype JL and HAP chemotype HAN. Metabolites analysis revealed that 72.18% of sesquiterpenes was highly accumulated in HAN compared to JL. Integrated omics analysis found a *DBR2-Like* (*DBR2L*) gene may be involved in artemisinin biosynthesis. DBR2L was highly homologous with DBR2*,* belonged to ORR3 family, and had the DBR2 activity of catalyzing artemisinic aldehyde to dihydroartemisinic aldehyde. Genome resequencing and promoter cloning revealed that complicated variations existed in *DBR2L* promoters among different varieties of *A. annua* and were clustered into three variation types. The promoter activity of diverse variant types showed obvious differences. Furthermore, the core region (−625 to 0) of the *DBR2L* promoter was identified and candidate transcription factors involved in *DBR2L* regulation were screened. Thus, the result indicates that DBR2L is another key enzyme involved in artemisinin biosynthesis. The promoter variation in *DBR2L* affects its expression level, and thereby may result in the different yield of artemisinin in varieties of *A. annua*. It provides a novel insight into the mechanism of artemisinin-producing difference in LAP and HAP chemotypes of *A. annua,* and will assist in a high yield of artemisinin in *A. annua*.

## Introduction

Artemisinin (Qinghaosu) and its derivatives (ARTs) are the first-line antimalarial drugs recommended by the World Health Organization [1]. They are widely recognized as having low toxicity, high bio-availability, and effective curative treatment for falciparum malaria infection [2, 3]. ARTs have been extensively studied for their efficacy in terms of anti-cancer, anti-inflammatory, immune regulation, etc. [4]. Recent studies reported that ARTs drugs could be effectively used for Coronavirus Disease 2019 (COVID-19) treatment [5]. Therefore, the demand for artemisinin-based drugs has been rapidly increased due to its application potential in the pharmaceutical industry. The traditional Chinese herb *Artemisia annua* L*.* (qinghao) is the only known plant source of artemisinin. Due to the low content of artemisinin in wild *A. annua*, many efforts have been attempted to produce more artemisinin through metabolic engineering in heterologous microorganisms and plants hosts [6, 7]. Nonetheless, breeding of superior cultivars of *A. annua* remains an important concern, because the high cost and technical difficulties made these genetic engineering methods unable to cover the supplement for global demand of artemisinins. In order to increase the production of artemisinin in *A. annua* and reduce its industrial extraction costs, it is necessary to further elucidate the artemisinin biosynthesis mechanism in *A. annua*.

The artemisinin biosynthesis pathway has been extensively but not fully elucidated. It includes the biosynthesis of sesquiterpene precursor farnesyl diphosphate and artemisinin specific biosynthesis pathway. The first step of artemisinin-specific biosynthesis is the synthesis of amorpha-4,11-diene (AD) by amorpha-4,11-diene synthase (ADS) from farnesyl diphosphate derived from both the mevalonate (MVA) and methylerythritol phosphate (MEP) pathways [8, 9]. Following the synthesis of AD, the other three key enzymes, cytochrome P450 monooxygenase (CYP71AV1) [10], artemisinic aldehyde Δ11 (13) reductase (DBR2) [11] and aldehyde dehydrogenase 1 (ALDH1) [12] sequentially synthesize artemisinic acid (AA) and dihydroartemisinic acid (DHAA). These products are conversed into artemisinin (ART), artemisinin B (AB), and artemisitene (ATT) in a nonenzymatic photo-oxidation manner [13]. Research on the regulation of artemisinin biosynthesis is also advancing and the related transcription factors (TFs) have been successively reported in recent years [14]. For instance, the glandular trichome development-related gene trichome and artemisinin regulator 1 (*AaTAR1*) [15], abscisic acid and drought response gene *AabZIP1* and jasmonic acid response gene *AaMYC2* were identified as positive regulators of artemisinin biosynthesis [16, 17]. The ethylene response gene ethylene-insensitive3 (*AaEIN3*) [18], and the AaMYC2 binding site competitors *AabHLH2* and *AabHLH3* were identified as negative regulators of artemisinin biosynthesis [19]. All these TFs modulate the accumulation of artemisinin by directly or indirectly interacting with the key enzymes of artemisinin biosynthesis. In addition, researchers investigated the mechanism of artemisinin biosynthesis and regulation from the different chemotypes of *A. annua*. The low artemisinin production (LAP) and high artemisinin production (HAP) chemotypes are two recognized varieties of *A. annua* that have different contents of artemisinin and its direct precursors [20]. Studies also released that more saturated 11,13-position structures, like ART, were found in the HAP chemotype. On the contrary, the LAP chemotype accumulated a high-level of unsaturated 11,13-position structures, such as ATT [13]. These metabolic differences between LAP and HAP chemotypes can be attributed to the differences in gene structure and transcriptional regulation. A positive correlation between artemisinin content and copy number of *ADS* was found in the latest research [21], which may be a reason for the increased synthesis of amorpha-4,11-diene in the HAP chemotype. Two types of CYP71AV1 participate in multiple oxidation steps but lead to a difference in oxidase function. The N-terminal of HAP-type CYP71AV1 lacks seven amino acids, which reduces the stability of its enzyme protein, and leads to the release of more substrates for DBR2 activity [22]. DBR2, as a key enzyme that functions by reducing the 11,13-position of artemisinic aldehyde (AAA) to dihydroartemisinic aldehyde (DHAAA), is an essential factor that influences the differences in artemisinin accumulation [23, 24]. Therefore, the differential expression of *DBR2* has always been a research focus. Previous research showed that the expression of *DBR2* in HAP was significantly higher than in LAP, and the promoter activity of *DBR2* may be an important factor influencing artemisinin yield. The large deletion in the *DBR2* promoter in LAP was thought to be one of the reasons for the difference in *DBR2* expression between LAP and HAP, indicating that was another reason for the different artemisinin-production between LAP and HAP chemotypes [23]. These studies have explained the regulation mechanism of artemisinin biosynthesis from different perspectives. However, the metabolite changes in the biosynthetic pathway of artemisinin have not been systematically explored and how *A. annua* varieties with different artemisinin contents are formed remains not fully understood.

Our research group has collected a large number of wild germplasms of *A. annua* from China (Table S1, see online supplementary material) and found that the artemisinin content of germplasm from the Hainan Province (HAN) was significantly higher than that from the Jilin Province (JL). Therefore, we chose JL as the LAP chemotype and HAN as the HAP chemotype in this study and systematically compared the genomic, metabolomic, and transcriptomic difference between them to further elucidate the mechanism of differential accumulation of artemisinin in LAP and HAP chemotypes of *A. annua.*

## Results

### Significant content changes of artemisinin-related compounds between JL and HAN

**Figure 1:**
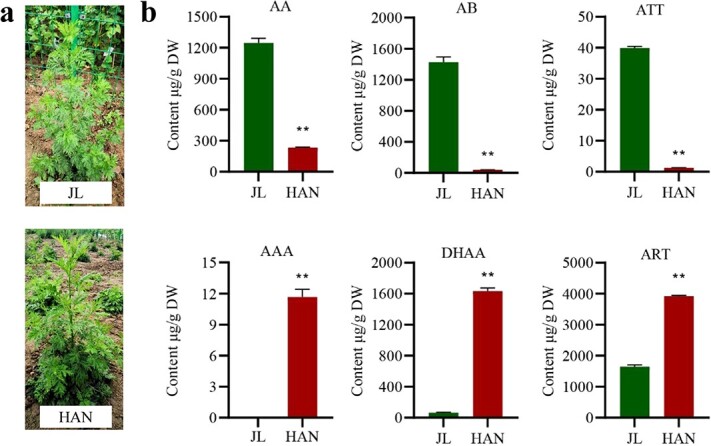
Content changes of artemisinin compounds between JL and HAN. **a** Phenotypes of JL and HAN. **b** Content changes of artemisinin compounds between JL and HAN. Data are means ± SD (*n* = 3). ^**^ indicate *P*-values <0.01. AA, Artemisinic acid; AAA, Artemisinic aldehyde; AB, Artemisinin B; ART, Artemisinin; ATT, Artemisitene; DHAA, Dihydroartemisinic acid.

Our observations showed there were no significant phenotypic differences between JL and HAN (Fig. 1a). Thus, the content changes of six artemisinin-related compounds in the artemisinin biosynthesis pathways between JL and HAN were determined using the optimized UPLC-QqQ(APCI)-MS/MS method (Fig. 1b). The results showed that AAA, DHAA, and ART were substantially higher in HAN than that in JL; specifically, the content of DHAA and ART in HAN was 25.36 and 2.38 times higher than that of JL, respectively. In contrast, the AA, AB, and ATT content was considerably higher in JL than in HAN; specifically, the content of AA and AB in JL was 5.32 and 36.77 times higher than that of HAN, respectively. This result indicates that JL and HAN were typical LAP and HAP chemotypes of *A. annua*, respectively. AAA, the common precursor of AA and DHAAA, was only detected in HAN, but not in JL, indicating that HAN accumulated more AAA. Moreover, due to the structural difference between ATT and ART at 11,13-position [13], the higher accumulation of ATT in JL indicated that the oxidation capacity of JL and HAN at this position was different.

### Genome resequencing exhibits sequence variations in the *DBR2L* promoter between JL and HAN

**Figure 2:**
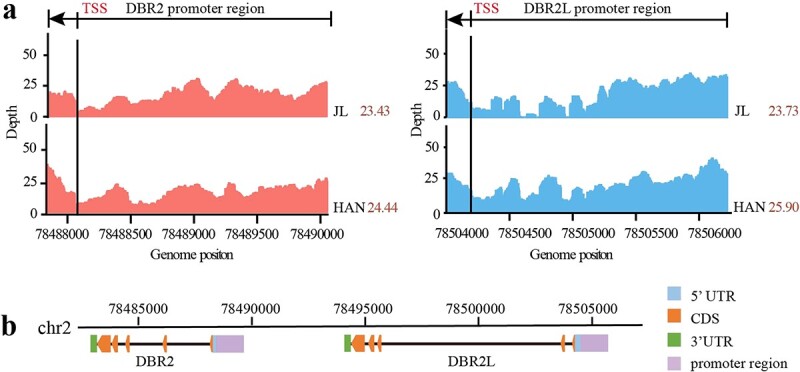
Sequence variations analysis of the *DBR2* and *DBR2L* promoter region by whole-genome resequencing. **a** The depth distribution analysis of the *DBR2* and *DBR2L* promoter region using whole-genome resequencing data of JL and HAN. **b** An illustration of the location of gene structure of *DBR2* and *DBR2L*.

*DBR2* is the checkpoint enzyme in the artemisinin biosynthesis pathway, and the sequence variations in the *DBR2* promoter region were considered as important factors affecting *DBR2* expression levels, thereby affecting artemisinin production [23]. Thus, the whole-genome resequencing data of JL and HAN (unpublished data) were mapped to the reference genome (LQ-9 phase 0) to analyse the sequence variations on the region near the *DBR2* gene. The results showed that there were significant differences in the read depth of coverage of JL and HAN, indicating sequence variations in the *DBR2* promoter region between JL and HAN (Fig. 2a), which is consistent with the literature report. Furthermore, the result found a gene (AA049700) adjacent to *DBR2*, which also exhibits significant sequence variations in its promoter region (Fig. 2a)*.*

This gene has been annotated as *DBR2* or *DBR2.1* in the two published versions of *A. annua* genome, but its function has not been reported yet. The gene location analysis showed that these two genes were distributed near each other on chromosome 2, and each had five exons (Fig. 2b). Nucleic acid sequence alignment revealed that this gene was a homolog of *DBR2*, with a 95% sequence similarity (Fig. S1, see online supplementary material). Therefore, we named it *DBR2-like* (*DBR2L*) and speculated that this gene was also involved in artemisinin biosynthesis.

### Volatile metabolomic and transcriptomic analysis of JL and HAN

GC–MS was utilized to investigate the differences in volatile metabolites between JL and HAN. A total of 1131 volatiles were putatively detected and the principal component analysis (PCA) plot revealed obvious differences between the JL and HAN groups (Fig. S2a, see online supplementary material). Among all the 133 identified sesquiterpenoids, 96 (72.18%) were accumulated more in HAN than in JL (fold change >1) (Fig. S2c, see online supplementary material). Meanwhile, 575 differentially accumulated metabolites (DAMs) between JL and HAN groups were identified of which 320 were upregulated and 255 were downregulated (Fig. S2b, see online supplementary material). Among these DAMs, terpenoids, phenol, ester, acid, aldehyde, and ketone were accumulated more in HAN, while alcohol, amine, hydrocarbons, and heterocyclic compounds accumulated more in JL. There were 266 terpenoids including 144 DAMs that were detected between JL and HAN, which contained 84 monoterpenes (43 downregulated, 41 upregulated), 59 sesquiterpenes (four downregulated, 55 upregulated), and one diterpene (one upregulated) (Fig. S2c, see online supplementary material). Thus, the result indicated that there were distinct differences in the volatile metabolites between JL and HAN, and more sesquiterpenes were accumulated in HAN.

**Figure 3:**
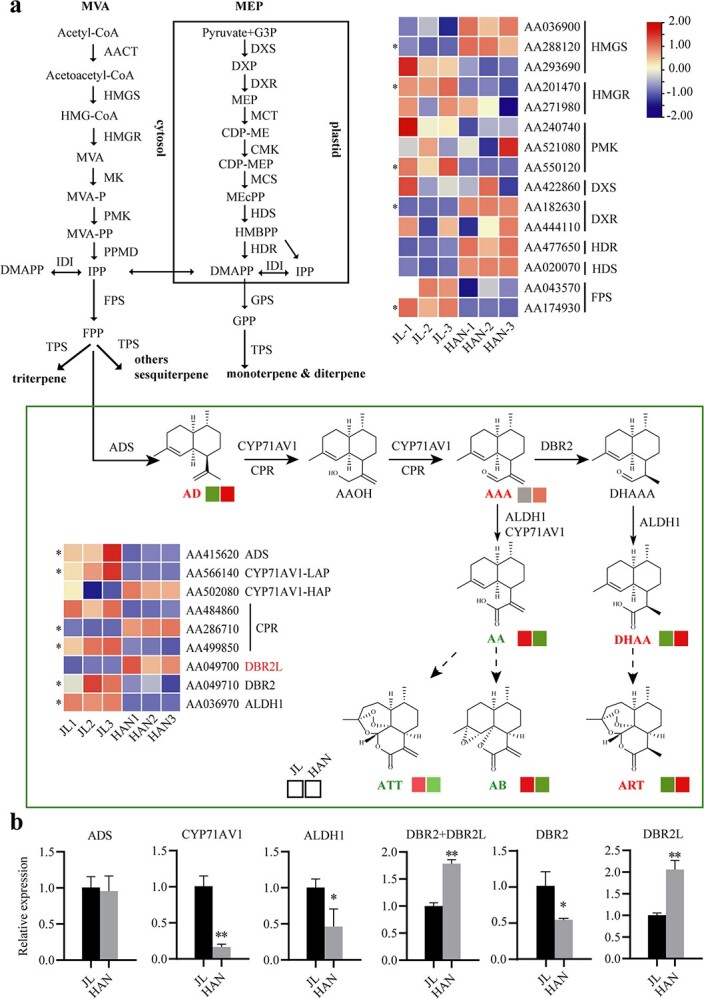
Differential analysis of artemisinin biosynthesis pathway between JL and HAN. **a** Integrative analysis of metabolites and genes involved in artemisinin biosynthesis. DEGs in the heatmap are indicated with *. AA, Artemisinic acid; AAA, Artemisinic aldehyde; AB, Artemisinin B; ALDH1, aldehyde dehydrogenase 1AACT, Acetoacetyl-CoA thiolase; ART, Artemisinin; ATT, Artemisitene ADS, amorpha-4,11-diene synthase; CDP-ME, 4-(cytidine- 5′-diphospho)-2-C-methyl-D-erythritol; CDP-MEP, 4-diphosphocytidyl-2C- methyl-D-erythritol 4-phosphate; CMK, 4-(cytidine-5′-diphospho)-2-C-methyl-D-erythritol kinase; CMT, 4-diphosphocytidyl-2C- methyl-D-erythritol 4-phosphate synthase; CPR, Cytochrome p450 Reductase; CYP71AV1, Cytochrome P450 Monooxygenase; DBR2, Artemisinic aldehyde Δ11 (13) reductase; DHAA, Dihydroartemisinic acid; DMAPP, dimethylallyl diphosphate; DXP, 1-Deoxy-D-xylulose 5-phosphate; DXR, 1-deoxy-D-xylulose-5-phosphate reductoisomerase; DXS, 1-deoxy-D-xylulose-5-phosphate synthase; FPS: farnesyl pyrophosphate synthase; GPP, geranyl diphosphate; G3P, glyceraldehyde 3-phosphate; HBMPP, 4-hydroxy- 3-methylbut-2-enyl diphosphate; HDR, 4-hydroxy-3-methylbut-2-enyl diphosphate reductase; HDS, 1-hydroxy-2-methyl-2-(E)-butenyl 4-diphosphate synthase; HMGR, HMG-CoA reductase; HMGS, 3-hydroxy-3-methylglutaryl-coenzyme A(HMG-CoA)synthase; IDI, isopentenyl-diphosphate isomerase; IPP, isopentenyl diphosphate; MCS, 2C-methyl-D-erythritol 2,4-cyclodiphosphate synthase; MEcPP, 2C- methyl-D-erythritol 2,4-cyclodiphosphate; MEP, 2C-methylerythritol 4- phosphate; MK, mevalonate kinase; PMK, phosphomevalonate kinase; PPMD, mevalonate-5-diphosphate decarboxylase. **b** qRT-PCR verification of key enzyme genes in artemisinin biosynthesis. Data are means ± SD (*n* = 3). ^**^indicate *P*-value <0.01; ^*^indicate *P*-value <0.05.

Transcriptome sequencing was performed using the same samples as the metabolic analysis. 39 939 genes were mapped according to the reference genome and their expression was analysed using PCA (Fig. S3a, TableS2, see online supplementary material). There were 11 235 differentially expressed genes (DEGs), of which 5833 were upregulated and 5402 were downregulated (Fig. 3b). Seventy Gene Ontology (GO) terms were significantly enriched (q < 0.05), of which the ‘biological process’ category with the highest enrichment (Fig. S4, see online supplementary material). Meanwhile, Kyoto Encyclopedia of Genes and Genomes (KEGG) analysis revealed that 20 DEGs (13 upregulated, seven downregulated) were enriched in sesquiterpene and triterpene synthesis pathways (Fig. S3c, see online supplementary material), indicating these genes may affect the differential accumulation of terpenoids in JL and HAN.

### Integrative analysis of metabolites and genes involved in terpenoids biosynthesis and artemisinin biosynthesis

To investigate the mechanism of differential accumulation patterns of artemisinin and sesquiterpenes between JL and HAN, we further conducted a comprehensive analysis of the difference of metabolites and gene expression. The heatmap of key enzyme genes in the terpenoid backbone biosynthesis pathway showed that *HMGS*, *DXR*, *HDR*, and *HDS* were highly expressed in HAN, while *HMGR*, *PMK*, and *FDS* were highly expressed in JL (Fig. 3a). Thus, the expression level of key enzyme genes in the terpenoid backbone biosynthesis pathway in HAN did not exhibit an obvious upregulated trend. Sesquiterpene synthases (*STSs*) are responsible for the synthesis of various sesquiterpene. All *STSs* in *A. annua* were annotated by blastN to investigate their expression profile (Fig. S5, see online supplementary material). A total of 51 *STS*s were identified from 93 terpene synthase (*TPS*) genes in *A. annua*. Thirty-eight *AaSTS*s were expressed in these transcriptomes, of which 16 were significantly differently expressed between JL and HAN (10 upregulated, six downregulated) (Fig. S6, see online supplementary material). This result indicated that *AaSTS*s had different expression patterns in JL and HAN, and high expression *AaSTS*s account for the majority in HAN, which is consistent with the accumulation profiles of sesquiterpenes in these two varieties.

For artemisinin biosynthesis, nine genes were involved in this pathway, of which three genes were upregulated and six were downregulated. *ADS* was not highly expressed in HAN either in the transcriptome data or by qRT-PCR verification (Fig. 3a and b). Previous studies also reported that there was no strong relationship between the expression difference of *ADS* and artemisinin yield [23]. The expression of *CYP71AV1-LAP* was significantly downregulated, while *CYP71AV1-HAP* was upregulated in HAN, indicating that more substrates are accumulated and released downstream in HAN. The expression of *ALDH1* was significantly downregulated in JL, which is consistent with the observed high accumulation of AA. It is noteworthy that we found that the expression of the previously identified *DBR2* (*AA049710*) was low expressed in both JL and HAN (FPKM value was 0.1–1.3) (Table S3, see online supplementary material), but the expression of *DBR2L* was significantly upregulated in HAN. Literature review found that these two genes were often confused due to their similar sequences and were usually not accurately distinguished in qRT-PCR assay (Table S4, see online supplementary material). Thus, we designed specific qRT-PCR primers to accurately quantify the expression levels of these two genes. The qRT-PCR results were consistent with transcriptome data (Fig. 3b), indicating that *DBR2L* was indeed significantly highly expressed in HAN than in JL. In addition, the expression levels of *DBR*2 and *DBR2L* examined in six tissues and organs of *A. annua* showed that *DBR2L* had a similar expression trend to *DBR2*, with both highly expressed in the bud and the upper leaves, and lowly expressed in root and stem (Fig. S7, see online supplementary material). The results further implied that *DBR2L* might be another key gene involved in artemisinin biosynthesis.

### Identification and functional analysis of DBR2L

**Figure 4:**
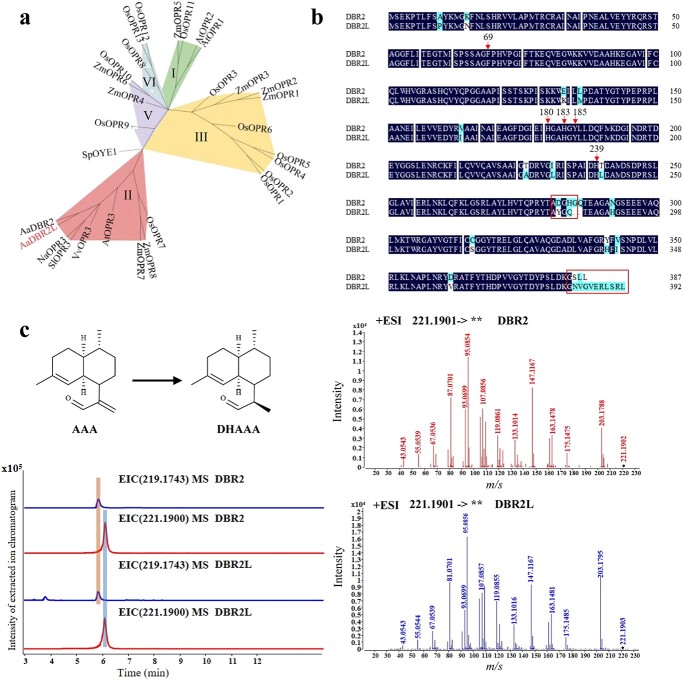
Identification and functional analysis of DBR2L. **a** The phylogenetic tree of DBRL and OPR family. The phylogenetic tree was generated by the neighbor-joining method using the MEGA7 with bootstrap values (1000 replicates). Aa, *Artemisia annua*; At, *Arabidopsis thaliana*; Na, *Nicotiana attenuata*; Os, *Oryza sativa*; Sl, *Solanum lycopersicum*; Vv, *Vitis vinifera*; Zm, *Zea mays.***b** Amino acid alignment of DBR2 and DBR2L. Arrows represent conserved sites. Boxes represent the main variation regions. **c** DBR2L catalyzes AAA into DHAAA. The extracted ion chromatogram (EIC) of AAA and DHAAA (the left figure), and the secondary mass of DBR2L products compared with that of DBR2 product (the right figure). AAA, *m*/*z* [M+H]^+^= 219.1743 ; DHAAA, *m*/*z* [M+H]^+^= 221.19 .

Based on the above results, *DBR2L* and *DBR2* are highly homologous, the *DBR2L* promoter region showed obvious sequence variations, and the gene expression levels of *DBR2L* were significantly different between JL and HAN. These findings suggested that DBR2L may also have similar functions to DBR2 and involve in differential accumulation of artemisinin in *A. annua*. Thus, we cloned *DBR2L* to verify its function. The open reading frame of *DBR2L* is 1179 bp and encodes a 392-amino acid protein. Nucleic acid sequence alignment showed the cloned *DBR2L* sequence was highly homologous to the reference sequence, except for a few single nucleotide polymorphisms (Fig. S8, see online supplementary material). Blastp comparison through NCBI revealed that this gene belongs to the 12-oxo-phytodienoic acid reductase (*OPR*) gene family. The phylogenetic tree was constructed by protein sequences including DBR2L, its homologous genes and OPR genes in Arabidopsis, rice, and maize (Fig. 4a). The results showed that DBR2 and DBR2L, OsOPR7 and AtOPR3, etc. clustered into group II of the OPR family. DBR2L has the highest homology with DBR2, with a protein sequence similarity of 95.58%, and also shares more than 75% homology with the OPR3 protein of tomatoes and grapes. The amino acid sequence alignment (Fig. 4b) revealed that DBR2L also contained the same OPR conserved amino acids as DBR2, inducing catalysis position His^180^, His^183^, His^185^ and the substrate binding cavity positions of His^239^ and Phe^69^_._ Their sequence differences were mainly two regions, one was ‘AYG’(A281-G283) in DBR2L and ‘ADGHG’ in DBR2 (A281-G285), the other was located in the C-terminal region. SLL in DBR2 was replaced in NVGVERLSRL.

The catalytic activity of DBR2L was verified through construction of its prokaryotic expression vector, with the identified DBR2 (EU704257.1) used as a positive control. The recombinant enzymes were purified from *Escherichia coli* cells and their protein sizes were detected by SDS-PAGE (Fig. S9, see online supplementary material). *In vitro* enzymatic assays confirmed that DBR2L can catalyse AAA into DHAAA, which had the same retention time and fragment ions as DBR2 reaction products detected by UPLC-qTOF(ESI) MS/MS (Fig. 4c). The result showed that, similar to DBR2, DBR2L also exhibited the activity of catalyzing AAA into DHAAA, suggesting that it also plays a positive role in artemisinin accumulation.

### The *DBR2L* promoter variations and transcriptional activity differences in various *A. annua* varieties

**Figure 5:**
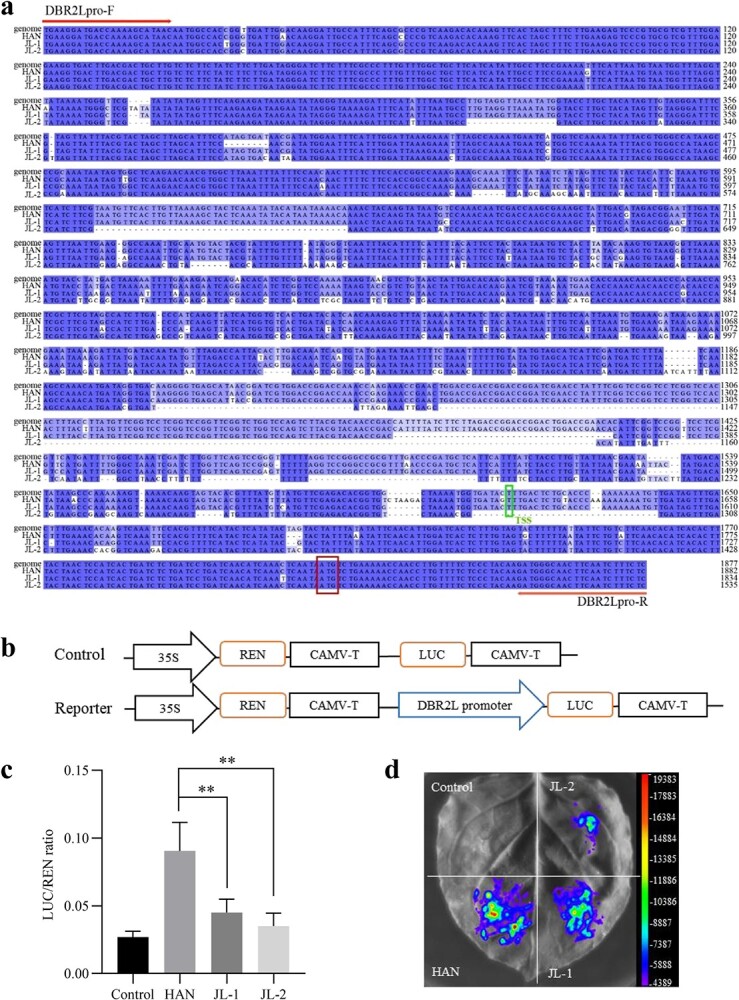
Promoter variations of *DBR2L* in JL and HAN and their activity analysis. **a** Comparison of the cloned *DBR2L* promoter in JL and HAN with LQ-9 phase 0 genome sequences. The cloning primers DBR2Lpro-F and DBR2Lpro-R were marked with arrow. Boxes represent the transcription starting site (TSS) based on LQ-9 phase 0 genome annotation and the starting codon of *DBR2L*. **b** Report construct schematic diagram used in the Dual-Luc assays. **c** Chemiluminescence determination of *DBR2L* promoter activity based on Dual-Luc assay system. Data are means ± SD (*n* = 3). **P-value <0.01. **d** Visualization of differences in *DBR2L* promoter activity by plant imaging system.

To verify the sequence variations, the promoters of *DBR2L* from JL and HAN varieties were cloned and sequenced. Three haplotypes were finally obtained, one from HAN (DBR2Lp-HAN, shorted for HAN), and two from JL (DBR2Lp-JL-1 and DBR2Lp-JL-2, shorted for JL-1 and JL-2), and the sequence lengths were 1883 bp, 1834 bp, and 1535 bp, respectively. Sequence comparison showed there were great variations between these three haplotypes (Fig. 5a). Among them, HAN was similar to the reference genome, while JL-1 and JL-2 had DNA fragment deletions. Thus, the sequence cloning results verified that there were sequence variations in the *DBR2L* promoter region. Moreover, the Dual*-*Luciferase (Dual-Luc) assay result demonstrated the Luc/Ren ratio in HAN was significantly higher than that in JL-1 and JL-2 (Fig. 5b–d), indicating the transcriptional activity of HAN is higher than that of JL-1 and JL-2. Thus, it can be concluded that the sequence variations of *DBR2L* promoters resulted in the difference of the promoter activity, thereby affecting the expression level of *DBR2L*.

**Figure 6:**
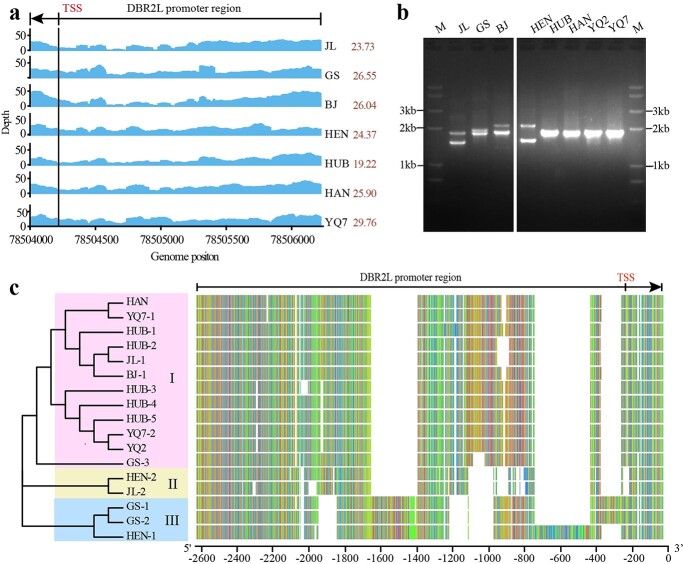
Promoter variations of *DBR2L* in various varieties of *A. annua*. **a** The depth distribution analysis of the *DBR2L* promoter region based on whole-genome resequencing data. The region 78 504 000 to 78 506 000 on chromosome 2 contains the *DBR2L* promoter and part of the gene sequence, and the transcription starting site (TSS) of the *DBR2L* was annotated based on reference genome LQ-9 phase 0. The average depth was marked on left. **b** PCR amplification of *DBR2L* promoters from different varieties of *A. annua*. M, molecular marker. **c** Phylogenetic analysis and sequence alignment of the cloned *DBR2L* promoters.

**Figure 7:**
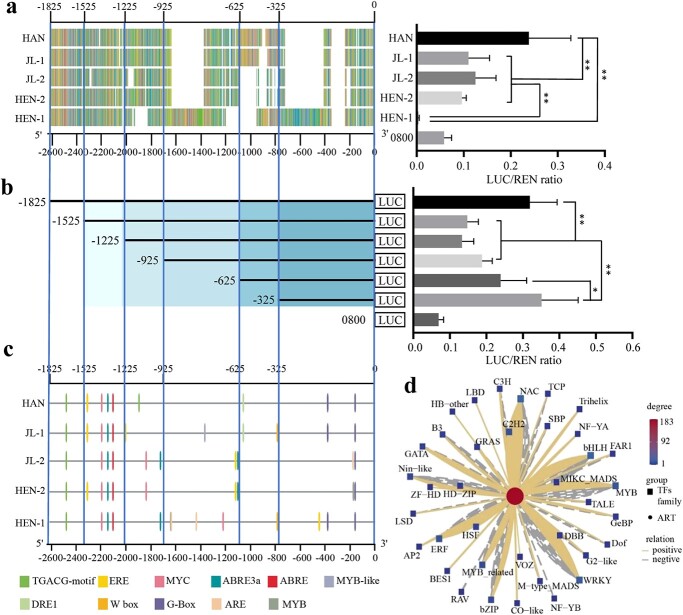
The core promoter region identification and candidate transcription factor screening of *DBR2L*. **a** Dual-Luc assay of different *DBR2L* promoter types. **b** Dual-Luc assay of progressive 5′ deletion constructs from −1825 to −325 of in pDBR2L-HAN. Data are means ± SD (*n* = 3). **P-value <0.01; *P-value <0.05. **c** Prediction of *cis*-acting regulatory elements of different *DBR2L* promoter types. **d** Correlation analysis between gene expression of differently expressed TFs and artemisinin content (Person R > |0.9| and *P*-value <0.05). ART, artemisinin.

To explore whether the sequence variations of the *DBR2L* promoter widely exist in various varieties of *A. annua*, we further analysed the depth of sequencing coverage in *DBR2L* promoter based on the whole-genome resequencing data of seven *A. annua* varieties, including six wild varieties – JL, GS, BJ, HEN, HUB, HAN – and one cultivated variety YQ7 (Table S1, see online supplementary material). The result indicated that sequence variations universally existed in the *DBR2L* promoter region in the tested varieties (Fig. 6a). Next, we cloned the promoter regions of *DBR2L* from these varieties. PCR result showed that a single band was amplified from HUB, YQ2, and YQ7 similar to HAN. Two bands were amplified from JL, GS, BJ, and HEN, but the size of these bands showed significant difference. Through PCR purification and clone-by-clone sequencing, a total of 17 sequences were obtained, and the length ranged from 1601 bp to 2142 bp. Phylogenetic tree analysis revealed the cloned sequences were clustered into three branches: type I, type II, and type III (Fig. 6b and c). All varieties except for HEN had a type I promoter, which was similar to HAN; HEN variety had type II promoters similar to JL-2; GS and HEN varieties also had the type III promoter, exhibiting greater sequence variations compared to types I and II. Although BJ amplified two bands, only one sequence belonged to type I promoter were obtained, the sequencing of the other band was failed due to special structure. However, because the position of the band in the gel electrophoresis was similar to HEN-1, it is speculated that this band should also belong to type III. Sequence alignment indicated there were obvious variations between the three sequence types. Among them, HAN and YQ7–1 had the same nucleic acid sequence, YQ7–2 and YQ2 had the same nucleic acid sequence, and the sequence similarity between these two haplotypes was 93%, indicating that the *DBR2L* promoters had little difference between artemisinin high-producing varieties. Moreover, sequences representing these three types of the *DBR2L* promoter, HAN, JL-1, JL-2, HEN-2, HEN-1 were screened for transcriptional activity analysis. The result showed that HAN exhibited the highest transcriptional activity, followed by JL-1, JL-2, HEN-2, but almost no luciferase activity was detected in HEN-1 (Fig 7a). It implied that the promoter variation was positively correlated with the level of transcriptional activity. Therefore, the *DBR2L* promoter had complex sequence variations between different varieties, which may regulate the expression of *DBR2L* by altering the transcriptional activity of the promoter, and thus affect the artemisinin biosynthesis in the different varieties.

As a representative of the *DBR2L* promoter in high artemisinin yield varieties, the sequence numbered HAN was selected to identify the critical regulatory regions in *DBR2L* promoter. A series of progressive 5′ deletion constructs of HAN promoter were fused to the luciferase reporter, then transiently transfected into *Nicotiana benthamiana* leaves. As demonstrated in Fig. 7b, the promoter construct p-1825 had higher luciferase activity, showed almost 4.5-fold transcriptional activity compared with the control vector, but the transcriptional activity of p-1525 significantly decreased, approximately 1/2 of that of p-1825. Further, the deletion of sequence from −1525 to −625 did not affect the *DBR2L* promoter activity; the deletion of sequence from −625 to −325 markedly increased the promoter activity, reached activity similar to p-1825. The result indicated that the sequence between −1825 and − 1525, and the sequence between −325 to 0 may be critical transcriptional activation regions in the *DBR2L* promoter, and the region from −325 to −625 may have silencers that inhibit the promoter activity.

Prediction of *cis*-acting regulatory elements (CREs) showed that the sequence variations between different haplotypes of the *DBR2L* promoter affect the types and quantity differences of transcription factor binding sites (TFBS) (Fig. 7c). In the transcriptional activation region from −325 to 0, HAN, JL-1, and HEN-1 contain double G-box (CACGTT), while JL-2 and HEN-2 only contain one G-box, but have an extra ARE (AAACCA) and MYB (CAACCA) site, respectively, both of which were the binding site of the MYB TF family. There are also differences in TFBS between different haplotypes in the region from −325 to −625. Only JL-1 and HEN-1 contain a WRKY family binding site W-box (TTGACC). Transcriptome analysis revealed that in total there were 198 TFs differentially expressed between JL and HAN (Fig. S10, see online supplementary material). Through the Pearson correlation analysis with artemisinin content (R^2^ > 0.9), 183 TFs related to artemisinin biosynthesis were screened, and the top five transcription families were MYB, NAC, bHLH, WRKY, and ERF (Fig. 7d), indicating these TF families may possibly participate in the regulation of *DBR2L* expression. This result provides candidate TFs involved in *DBR2L* regulation.

## Discussion

Sesquiterpenoids are the most complex terpenoids and play vital roles in plant physiological processes [25]. Artemisinin is a representative sesquiterpenoid that is extracted from *A. annua*. Artemisinin and its derivatives are currently considered to be the most effective drugs to treat malaria, and have therapeutic potential for future cancer treatment [26]. In addition, *A. annua* contains other sesquiterpenoids, such as β-caryophyllene, *epi*-cedrol, and β-farnesene [27–30]. In this study, two wild varieties of *A. annua*, JL and HAN, were used to compare the differences of artemisinin and sesquiterpenoids between them and investigate the possible molecular regulation mechanism. The UPLC-QqQ(APCI)-MS/MS analysis determined that AAA, DHAA, and ART were considerably higher in HAN, while AA, AB, and ATT were considerably higher in JL, indicating they were typical LAP and HAP chemotypes of *A. annua*, respectively*.* Volatile metabolomic analysis indicated that the HAP chemotype HAN not only accumulated artemisinin, but accumulated more sesquiterpenoids compared with the LAP chemotype JL, which improved our understanding of the differences in sesquiterpene between LAP and HAP chemotypes of *A. annua*. Transcriptome analysis results showed that 16 *AaSTS*s were differentially expressed, and 10 of 16 *AaSTS*s were significant upregulated in HAN than JL, which was consistent with the accumulation trend of sesquiterpenes.

DBR2 is the key enzyme in artemisinin biosynthesis and has 11,13-position reducing activity to catalyze AAA into DHAAA [11]. In this study, a comprehensive analysis of genome resequencing, metabolome, and transcriptome analysis of JL and HAN varieties revealed that *DBR2L* promoter region exhibited obvious sequence variations (Fig. 2a), and the expression of *DBR2L* was high in HAN (Fig. 3b), suggesting that *DBR2L* may be involved in artemisinin biosynthesis. Through searching of previous research literature and the NCBI database, it was found that DBR2L was annotated as DBR2 or DBR2.1 in the published *A. annua* genomes [21, 31], and identified as an OPR3 gene due to its high homology with *AtOPR3* in Arabidopsis. However, there was no report on the function of DBR2L. Our result showed that DBR2L belonged to group II of the OPR family, and had the highest homology with DBR2. However, DBR2L contains conserved motif ‘AYG’ (A281-G283) like AtOPR3, which were occupied by ‘ADGHG’ in DBR2. This motif was considered be related to the substrate specificity of OPRs [32]. Additionally, the C-terminal of amino acid residue in DBR2L (SRL) is consistent with that of AtOPR3, suggesting that DBR2L may also be localized to the peroxisome. The functional verification results showed that DBR2L could also catalyze AAA to form DHAAA (Fig. 4c), which confirmed that it had the same catalytic activity as DBR2 and demonstrated that DBR2L may be another key gene in artemisinin biosynthesis. Combining this result with its high expression in HAN, we can speculate that the high expression of *DBR2L* may be the reason for the increased artemisinin biosynthesis in the HAP chemotype HAN.

Promoter plays an important role in gene expression and regulation, and promoter variation is closely related to gene expression and thereby affects plant growth, development, and metabolism [33–36]. Studies have shown that the promoter sequences of *ADS*, *CYP71AV1*, and *ALDH1* in artemisinin biosynthesis were relatively similar, but the promoters of *DBR2* were different between *A. annua* varieties, which was presumed to be closely related to the differential expression of *DBR2* [23, 37]. However, the sequence variations of the *DBR2L* promoter have never been analysed. Our genome resequencing analysis and sequence cloning data indicated there were indeed obvious sequence variations in the *DBR2L* promoter between JL and HAN (Figs 2a and5a). The Dual-Luc assay indicated the promoter activity in HAN was stronger than that in JL (Fig. 5c–e). Thus, the difference in the promoter activity affects the expression level of *DBR2L* in JL and HAN, which may change the artemisinin production in these two varieties. The analysis of sequence variations in *A. annua* varieties showed that sequence variations in the *DBR2L* promoter were widely present in different varieties of *A. annua* (Fig. 6a–c)*.* These sequence variations were complicated and clustered into three types. Combined with the previously determined artemisinin content (Table S1, see online supplementary material) in different varieties, we found that these promoter variations in different varieties seemed to have a correlation with the artemisinin yield (Fig. 7a). Extracting five sequences from three types for activity analysis revealed the order of activity from high to low was: HAN > JL-1, JL-2, HEN-2 > HEN-1. The varieties with high artemisinin content like HAN had type I promoters, while the varieties that had lower artemisinin content like JL and HEN had type II and type III promoters. These two types occurred obvious DNA sequence insertions and deletions compared to type I. 5′ deletion constructs of the *DBR2L* promoter of HAN indicated that the sequence from −1825 and −1525 and that from −325 to 0 may be critical transcriptional activation regions in the *DBR2L* promoter, and the region from −325 to −625 may have silencers that inhibit the promoter activity. Combining the results of sequence variations and activity analysis of different variant types in Fig. 7a, it can be concluded that the sequence between −625 to 0 may be the core region associated to the transcriptional level of *DBR2L* in *A. annua* varieties. Finally, we predicted the changes of CREs caused by sequence variations in the *DBR2L* promoter and screened differentially expressed MYB, NAC, bHLH, WRKY, and ERF family TFs that may possibly participate in the regulation of *DBR2L* expression (Fig. 7c–d), which provides candidate TFs for further research on *DBR2L* regulation. Thus, the results suggest that the variation polymorphisms in *DBR2L* promoter may enable *DBR2L* to respond to stress [38, 39] and participate in complex transcriptional regulatory networks [40], which ultimately controls the expression level of *DBR2L* [41, 42], and thus affects the artemisinin biosynthesis in different varieties.

In conclusion, we identified a *DBR2L* gene*,* which had the same function as *DBR2* in artemisinin biosynthesis. Moreover, the complicated variations of the *DBR2L* promoters between the *A. annua* varieties resulted in changes in the activity of their promoters, thereby regulating the expression levels of *DBR2L* and affecting the artemisinin biosynthesis. Thus, the differential expression of *DBR2L* due to the promoter variation may be a novel factor that affects artemisinin biosynthesis in different varieties of *A. annua*. The finding provides a novel insight into the mechanism of artemisinin-production differences between the LAP and HAP chemotypes of *A. annua.* Furthermore, the core promoter region of *DBR2L* was identified and candidate TFs involved in *DBR2L* regulation were screened, which is helpful for further study.

## Materials and methods

### Plant materials and chemicals

*A. annua* varieties were collected by our group and the collection information was shown in Table S1 (see online supplementary material). Whole leaves of four-month-old plants were harvested in the planting base in Huairou District, Beijing, China for metabolites analysis and RNA sequencing. Six tissues and organs of HAN variety cultivated in a greenhouse (including the root, the stem, the upper leaves, and the lower leaves from three-month-old plants, and the flower and bud from four-month-old plants) were collected for gene expression pattern analysis. Each sample above has three independent replicates. Seedlings of *N. benthamiana* were greenhouse cultivated at 24°C (16-h light/8-h dark) to four weeks old. Chemical standards applied in this study were Artemisinic acid (ChemFaces, Wuhan, China; CFN97276), Dihydroartemisinic acid (ChemFaces, Wuhan, China; CFN93295), Artemisinin (ChemFaces, Wuhan, China; CFN99011), Artemisinin B (ChemFaces, Wuhan, China; CFN98807), Artemisitene (Toronto Research Chemicals, Toronto, Canada; A777550), and Artemisinic aldehyde (Toronto Research Chemicals, Toronto, Canada; A777510).

### Extraction and determination of artemisinin-related compounds

The lyophilization leaves of JL and HAN were ground into powder, added 2.5 ml methanol per 50 mg sample powder, 15 min (40 kHz) with ultrasonic extraction. The original supernatant was used to determine AAA and ATT, and the supernatant diluted by 100 times was used to determine DHAA, AA, AB, and ART. All extracts were filtrated by 0.22 μm dimension micropore film then analysed using a 1290–6470 UPLC-QqQ MS/MS system (Agilent, Santa Clara, California, USA). Analytical conditions in UPLC: column, Eclipse Plus C18, RRHD (2.1 $\times$ 50 mm, 1.8 μm); solvent system, phase A is ultrapure water (0.1% formic acid and 5 mM ammonium formate), phase B is methanol; gradient program, 0 min(55% B), 8 min (100% B), 11 min (100% B), 14 min (55% B); flow rate, 0.6 ml/min; temperature, 40°C. Mass data acquisition was performed in APCI positive polarity and MRM mode using the following parameters: dry gas temperature, 300°C; temperature of evaporation chamber, 400°C; dry gas flow rate, 4 L/min; atomizer pressure, 50 psi; corona needle current, 4 μA; capillary voltage, 2500 V. (The ionic fragment and standard curve are shown in Tables S5 and S6, see online supplementary material).

### Sequence variations of promoter regions by whole genome resequencing

The genome resequencing data of JL, HAN, and other *A. annua* varieties (unpublished) were mapped to the LQ-9 phase 0 reference genome [21], and the read depth in 2 kb of the upstream promoter region of *DBR2* and *DBR2L* genes were counted. The average read depth was calculated with 20 bp as a window and mapped with GGPlot2.

### Volatile metabolomic analysis

Freezing samples were ground into powder and the detection process was performed as previous studies [43]. Volatile metabolomes were performed by MetWare Biotechnology Co., Ltd (Wuhan, China). Ion monitoring (SIM) mode was used for identification and quantification. The MWDB database was used to identify volatile metabolites by m/z values, retention time, and fragmentation mode. DAMs were determined by VIP ≥ 1 and |log_2_fold change | ≥ 1.

### RNA sequencing

Total RNA was isolated by Magnetic Plant RNA Kit (Tiangen, Beijing, China) and assessed for integrity and concentration. RNA-seq was performed on an Illumina platform by Annoroad Gene Technology Co., Ltd (Beijing, China). These filter clean data were aligned to the *A. annua* genome [31, 44] (PRJNA416223) using HISAT2 v2.1.0. and read counts for each gene were counted by HTSeq v0.6.0. Genes with q (adjusted *P*-value) ≤ 0.05 and |log2_ratio| ≥ 1 were identified as DEGs. GO (http://geneontology.org/) and KEGG (http://www.kegg.jp/) database were used to annotate all DEGs. GO terms with q < 0.05 were significantly enriched and KEGG terms with *P*-value <0.05 were significantly enriched.

### Statistical analyses

PCA and volcano plots were prepared using Metware Cloud (https://cloud.metware.cn). Heatmap was generated and visualized by TBtools. Pearson’s correlation was calculated using the OmicStudio platform (https://www.omicstudio.cn/tool). Statistical analysis and significance *t*-tests were performed using GraphPad Prism 8. Chemical structural formula was drawn by ChemDraw 18.0.

### Identification and phylogenetic analysis of *AaTPS* family

AaTPS genes were identified by hmmsearch 3.1b2 (e-value <1^e-5^) with the Terpene_synth domain (PF01397) and Terpene_synth_C domain (PF03936) which were downloaded from Pfam (http://pfam.xfam.org/). Then all candidate TPSs were annotated by NCBI Nucleotide collection (Nr/Nt) database and compared by ClustalW and MEGA7. The phylogenetic tree was constructed by the neighbor-joining method (Bootstrap repeats = 1000) and visualized by Evolview website (https://www.evolgenius.info//evolview/).

### qRT-PCR analysis

RNA samples were synthesized into cDNA and analysed by qRT-PCR according to previous research procedures [45]; each sample, respectively, in three independent experiments. The primer information was shown in Table S7 (see online supplementary material).

### Cloning, phylogenetic analysis, protein expression, and enzyme assays of DBR2L

The coding DNA sequences (CDSs) of *DBR2L* were cloned from cDNA of JL and the sequence deposited in Genbank (accession number: OQ923388). The OPR family proteins in *Oryza sativa*, *Arabidopsis thaliana*, and *Zea mays* were downloaded from the respective databases according to Nie *et al*’s report [46]. Other homologous OPR3 genes were: AaDBR2 from *A. annua* (accession number: EU704257); NaOPR3, from *Nicotiana attenuata* (accession number: XP_019256011.1); VvOPR3 from *Vitis vinifera* (accession number: NP_001267975.1); SlOPR3 from *Solanum lycopersicum* (accession number: NP_001233873.1)*.* Sequence alignment and phylogenetic tree analysis were performed as above.

For protein expression, *DBR2* (accession number: EU704257) was synthesized by Tsingke Biotechnology Co., Ltd (Beijing, China). Both *DBR2* and *DBR2L* genes were fused to the pET-32a vector then transformed into *E. coli* BL21 (DE3). The primers sequences are listed in Table S7 (see online supplementary material). Protein expression was induced with 0.5 mM IPTG for 24 h at 16°C. Cells were collected and lysed by sonification for 10 min at 40 W. The soluble proteins were purified by His-tag Protein Purification Kit (Beyotime, Shanghai, China) and concentrated through ultrafiltration tubes (30 kDa; Millipore, Burlington, Massachusetts, USA), then identified by SDS-polyacrylamide gel electrophoresis.

The catalytic activity of DBR2 and DBR2L was verified using artemisinic aldehyde as substrates. The standard reaction mixture contained phosphate buffered saline, 1 mM NADPH, 0.5 mM substrates and purified protein. The reaction was performed at 30°C for 30 min and stopped by adding methanol. The catalytic reaction products were detected by 6530 UPLC-qTOF-MS/MS system (Agilent, Santa Clara, California, USA). Analytical conditions in UPLC: column, Poroshell 120, EC-C18 (2.1 $\times$ 50 mm, 1.9 μm); solvent system, phase A is ultrapure water (0.1% formic acid), phase B is acetonitrile; gradient program, 0 min(50% B), 8 min (100% B), 10 min (100% B); temperature, 30°C; flow rate, 0.3 ml/min. Mass data acquisition was performed in ESI positive polarity and auto MS/MS mode (MS Range 100–500 m/z, MS/MS Range 30–500 *m/z*) using the following parameters: dry gas temperature, 300°C; VCap, 3500 V; Fragmentor, 100 V; dry gas flow rate, 8 L/min.

### Cloning and sequence variations analysis of *DBR2L* promoter

The genomic DNA of eight *A. annua* varieties were extracted using DNeasy Plant Pro Kit (Qiagen, Germany), and the promoter region of *DBR2*L was amplified with primers DBR2Lpro-F/R (Table S7, see online supplementary material). For sequencing, the purified PCR products were cloned onto pMD18T vector and transformed into *E. coli* DH5α. Sequence alignment and phylogenetic analysis were analysed using MEGA7 and Jalview software.

### Dual-Luc assay of promoter activity of *DBR2L*

The promoter regions of *DBR2L* from JL, HEN, HAN, and a series of progressive 5′ deletion promoters of HAN were fused to the pGreenII 0800-Luc vector, and then transformed into *Agrobacterium tumefaciens* strain EHA105 together with the plasmid pSoup. Empty pGreenII 0800-Luc reporter was served as a negative control. EHA105 strains were infiltrated into *N. benthamiana* leaves referring to previous methods [47]. The leaves were collected after 48 h cultivation, then part of the sample was measured by the Dual-Luciferase Reporter Assay System (Promega, Madison, Wisconsin, USA). The other part of the samples was sprayed with 0.3 mg/ml D-luciferin potassium salt and detected by Vivo Plant Imaging System (Nightshade lb 98; Berthold, Schwarzwald, Germany).

## Supplementary Material

Web_Material_uhad164

## Data Availability

17 cloned *DBR2L* promoter sequences have been submitted to NCBI by BankIt tool; the accession numbers are OR268936-OR268940, OR348401-OR348410, OR354319-OR354320. The bioproject number of RNA sequencing data is PRJNA924664.
