# Supplementary material for: Promoter variations in DBR2-like affect artemisinin production in different chemotypes of Artemisia annua
Source: Hortic Res. 2023 Aug 16;10(9):uhad164. doi: 10.1093/hr/uhad164 (PMC10508037; doi:10.1093/hr/uhad164)
Supplement: Web_Material_uhad164 [file web_material_uhad164.docx]

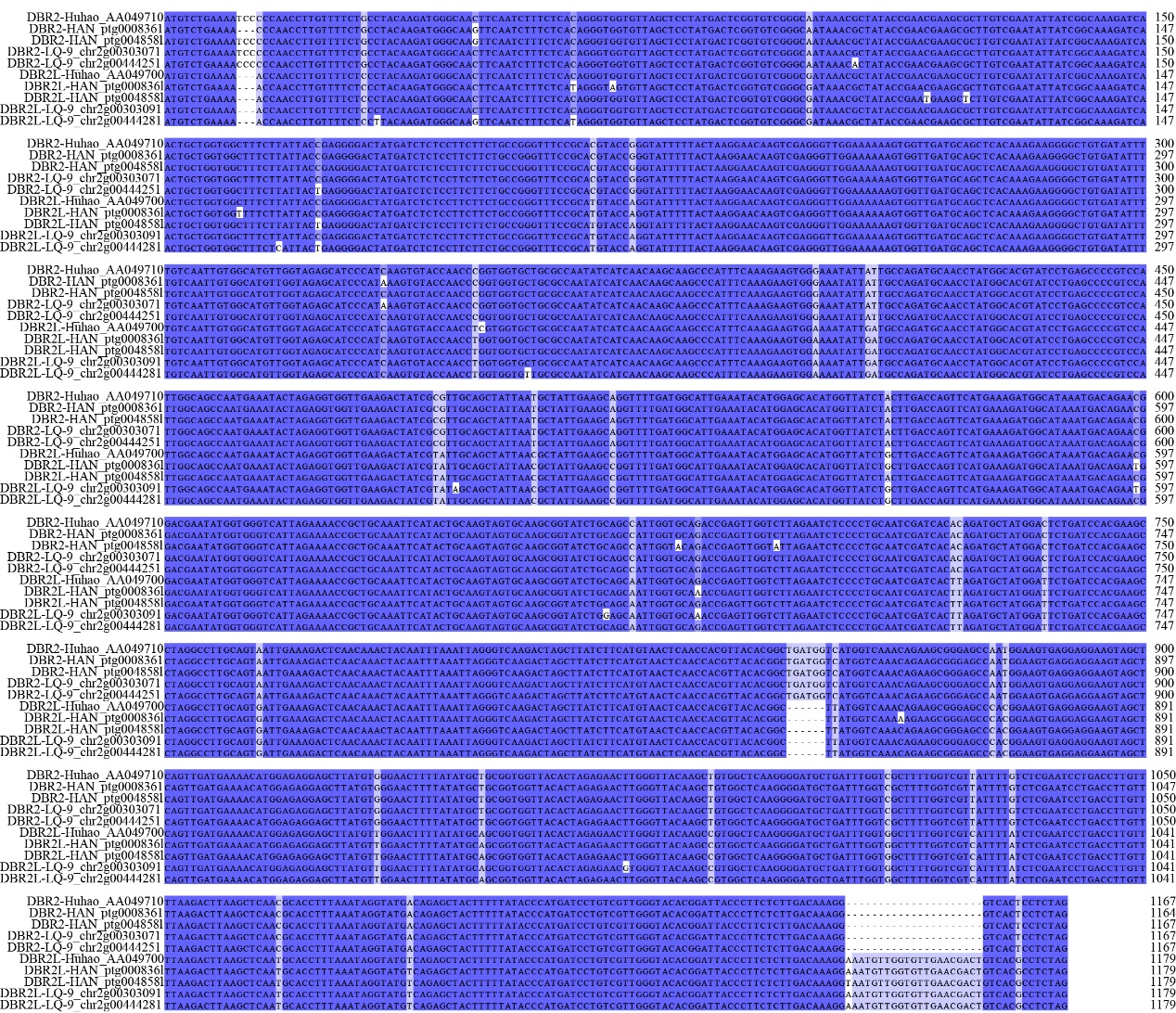


**Supplementary Figure S1 Comparison of nucleic acid sequences between *DBR*2 and *DBR2L***


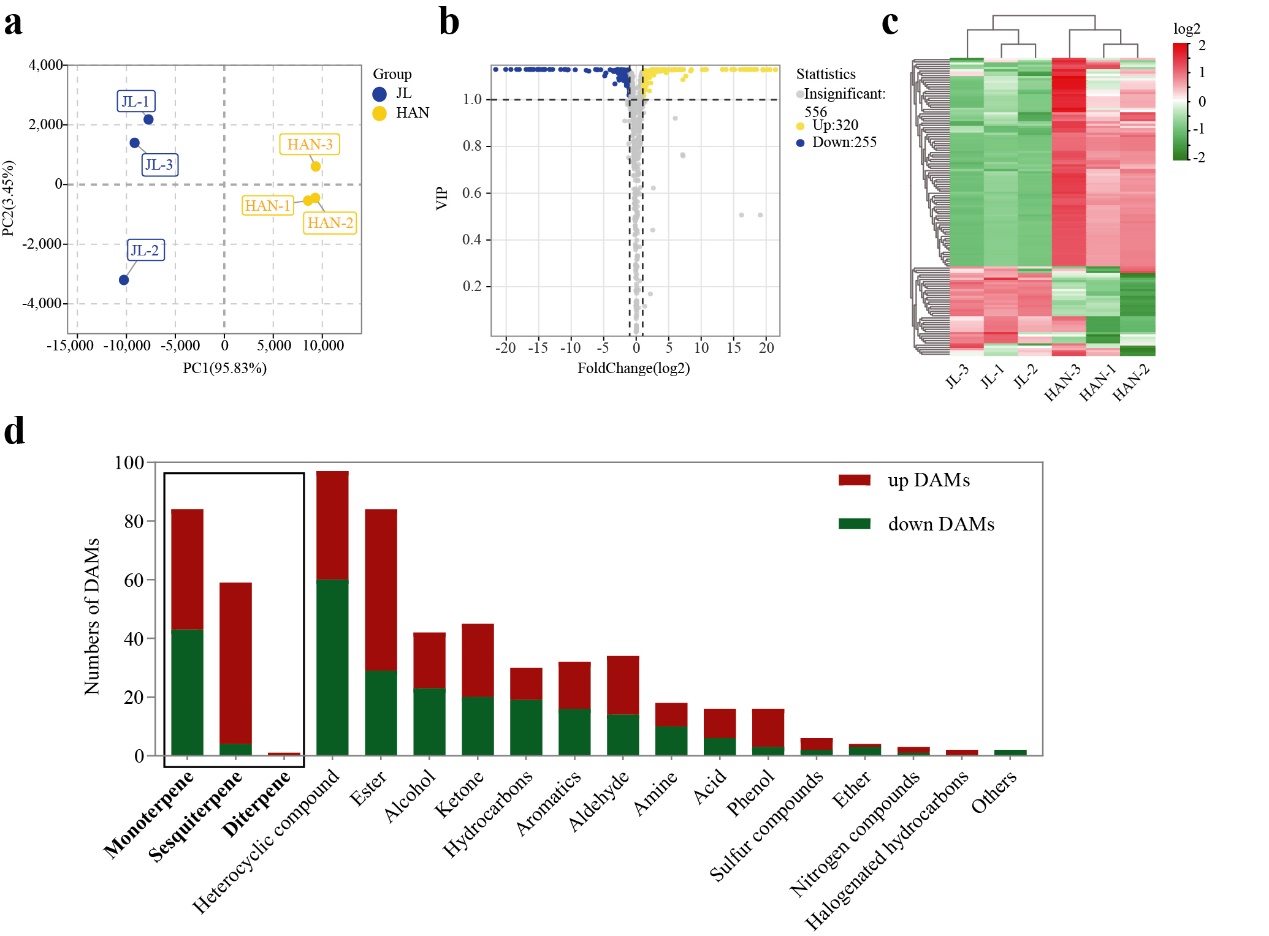


**Supplementary Figure S2 Analysis of volatile metabolome between JL and HAN. a** PCA analysis of DAMs between JL and HAN. **b** Volcano plot of DAMs between JL and HAN. **c** Sesquiterpene accumulation profiles of sesquiterpenes between JL and HAN. **d** Analysis of distribution of DAMs in various compounds between JL and HAN. Terpenes are marked with black box.


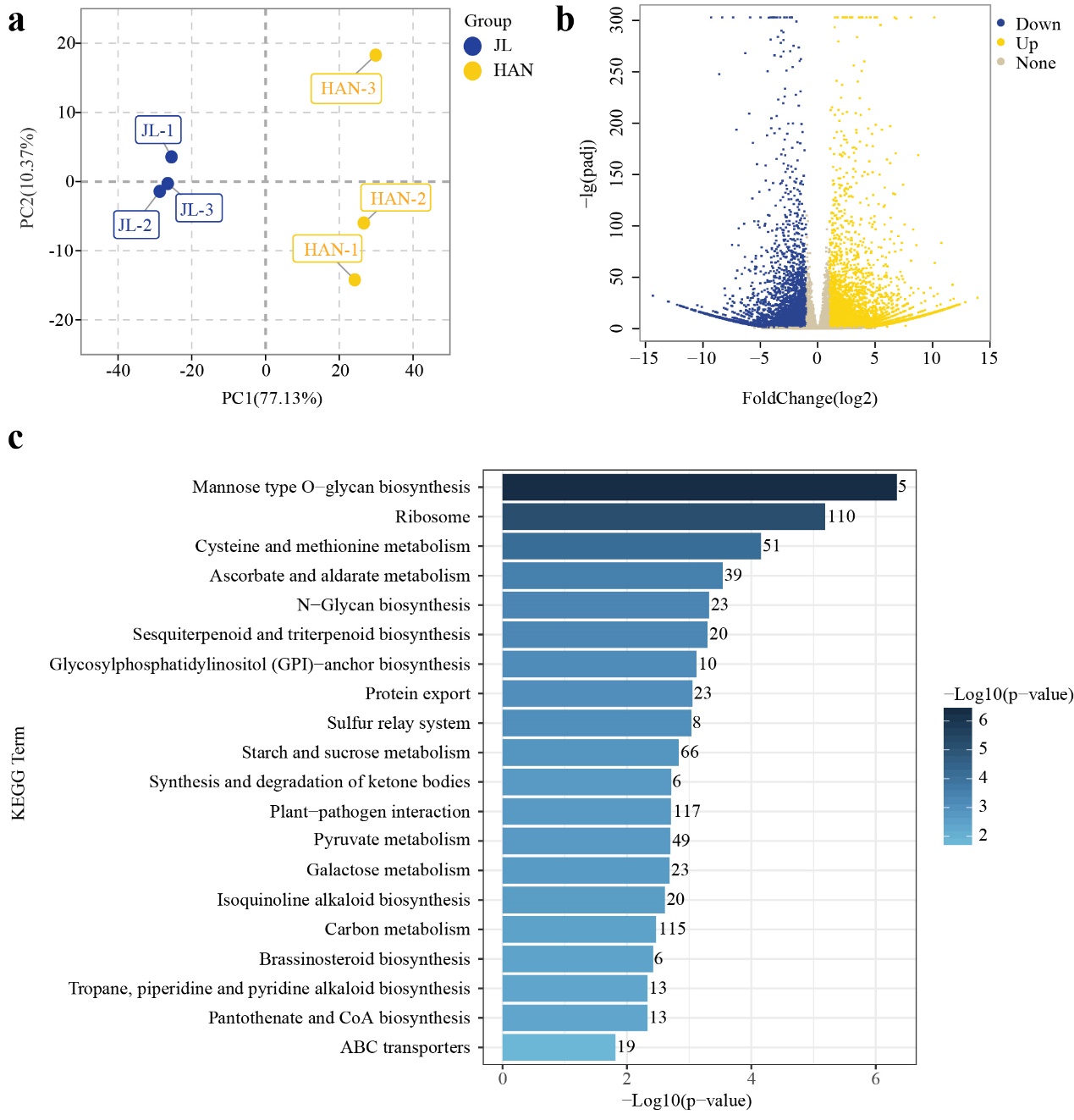


**Supplementary Figure S3 Analysis of transcriptome between JL and HAN. a** PCA analysis of DEGs between JL and HAN. **b** Volcano plot of DEGs between JL and HAN. **c** KEGG enrichment analysis of DEGs. 20 KEGG pathways with the most significant enrichment of DEGs are presented. The color depth indicates the significance of enrichment, and the number of genes enriched by terms is marked after.


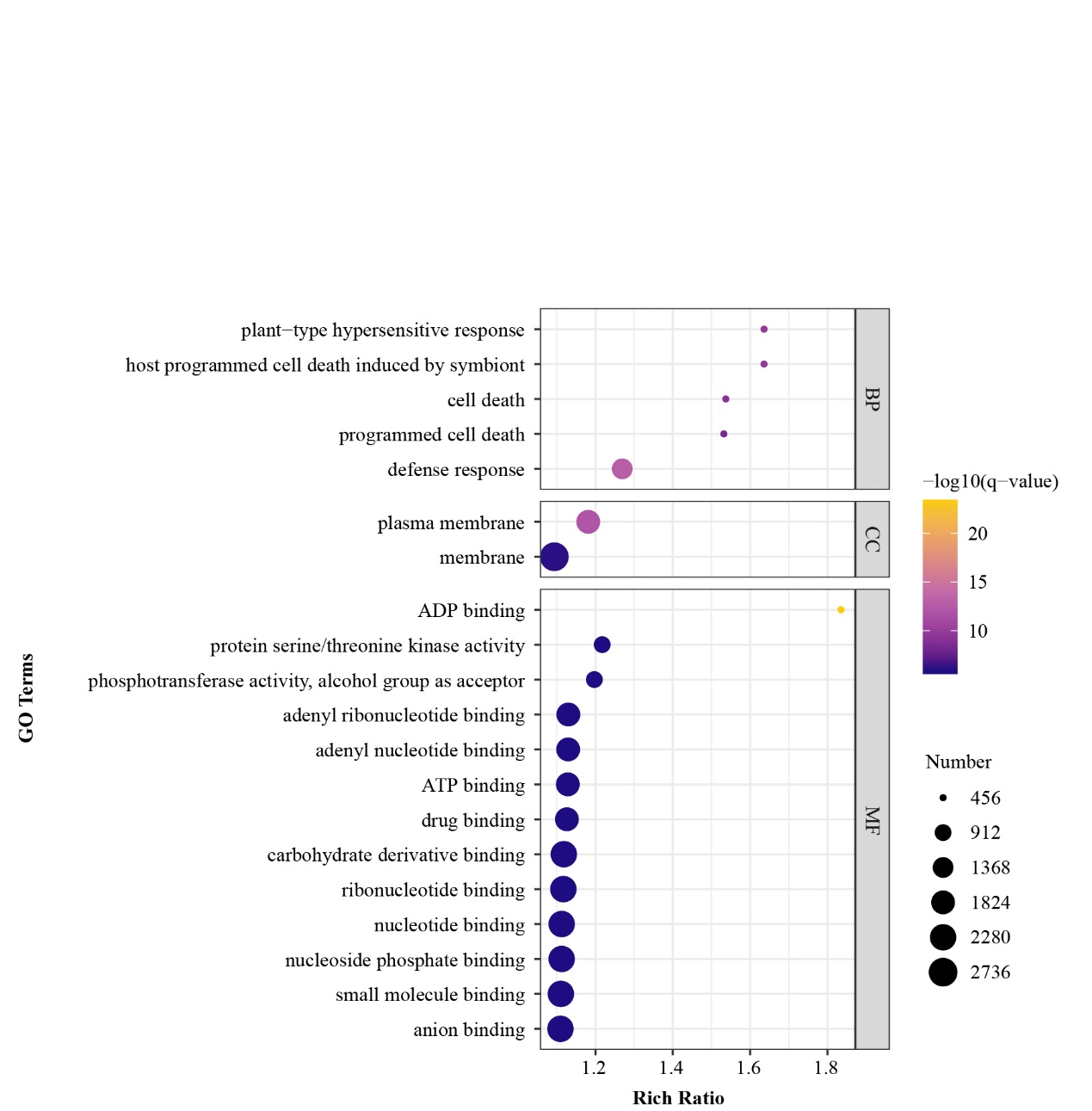


**Supplementary Figure S4 GO enrichment analysis of DEGs between transcriptome in JL and HAN.** The top 20 enriched GO terms are presented. The horizontal axis represents the rich factor, while the vertical axis represents the GO terms. Number: DEGs number; q-value: adjusted p-value.


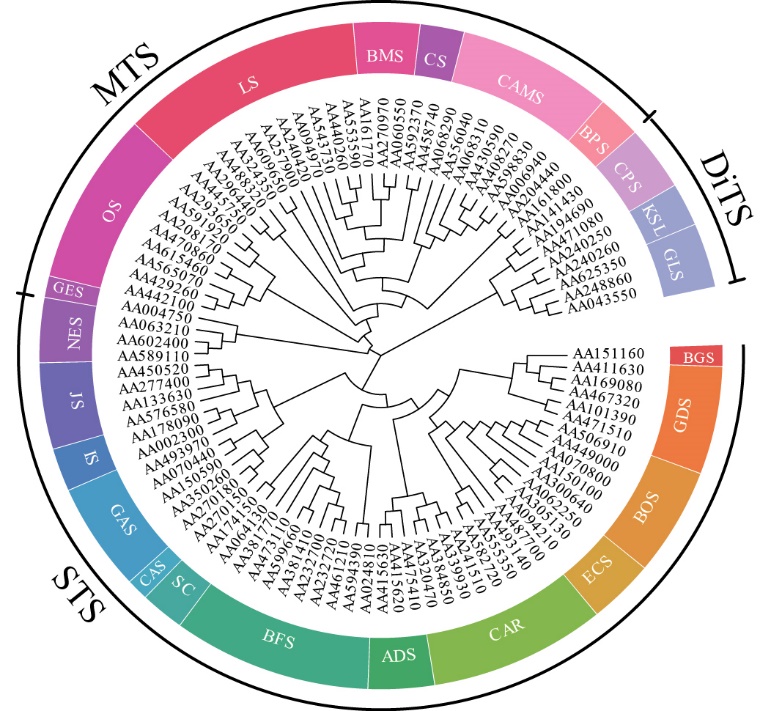


**Supplementary Figure S5 Functional characterization of terpene synthases in *A. annua*.** The protein sequences of 93 candidate terpene synthases were used to construct a Neighbor Joining tree with 1000 bootstrap replicates. BGS, bicyclogermacrene synthase; GDS, germacrene D synthase; BOS, alpha-bisabolol synthase; ECS, epi-cedrol synthase; CAR, beta-caryophyllene synthase; ADS, amorpha-4,11-diene synthase; BFS, E-beta-farnesene synthase; SC, sesquiterpene cyclase; CAS, cascarilladiene synthase; GAS, germacrene A synthase; IS, intermedeol synthase; JS, 10-epi-junenol synthase; NES, (E)-nerolidol synthase; GES, geraniol synthase; OS, (E)-beta-ocimene synthase; LS, linalool synthase; BMS, beta-myrcene synthase; CS, 1,8-cineole synthase; CAMS, camphene synthase; BPS, beta-pinene synthase; CPS, copalyl diphosphate synthase; KSL, kaurene synthase-like; GLS, geranyllinalool synthase; MTS, monoterpene synthase; STS, sesquiterpene synthase; DiTS, diterpene synthase.


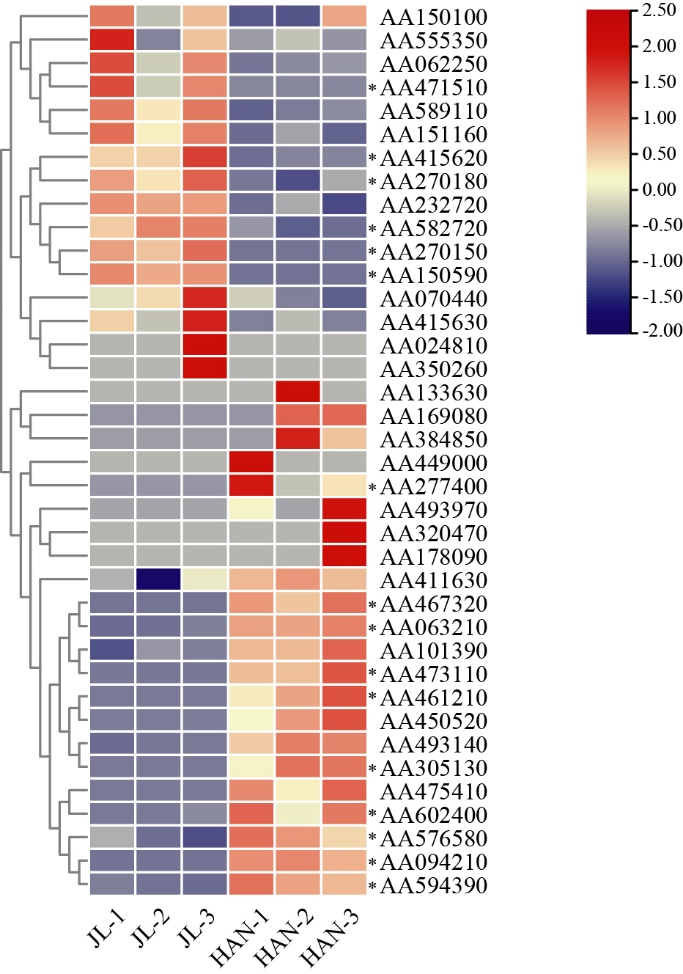


**Supplementary Figure S6 Expression profiles of candidate sesquiterpene synthases in JL and HAN**. DEGs are indicated with *.

**
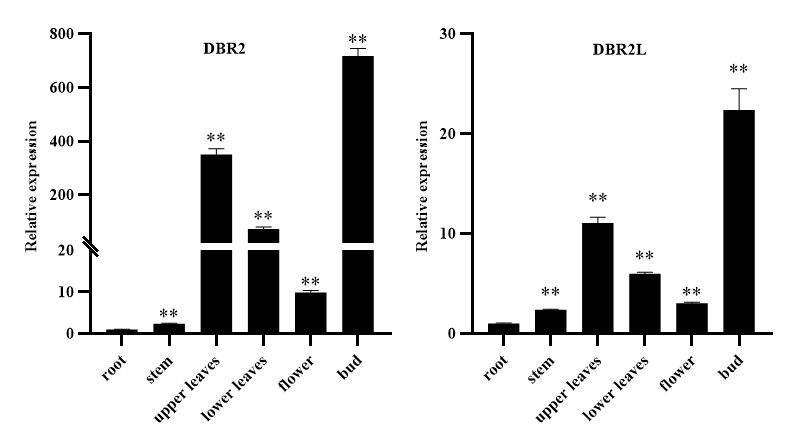
**

**Supplementary Figure S7 Expression patterns of *DBR2* and *DBR2L* in different tissues and organs in *A. annua.*** Asterisks indicate statistically significant differences compared with the root (**, p < 0.01).


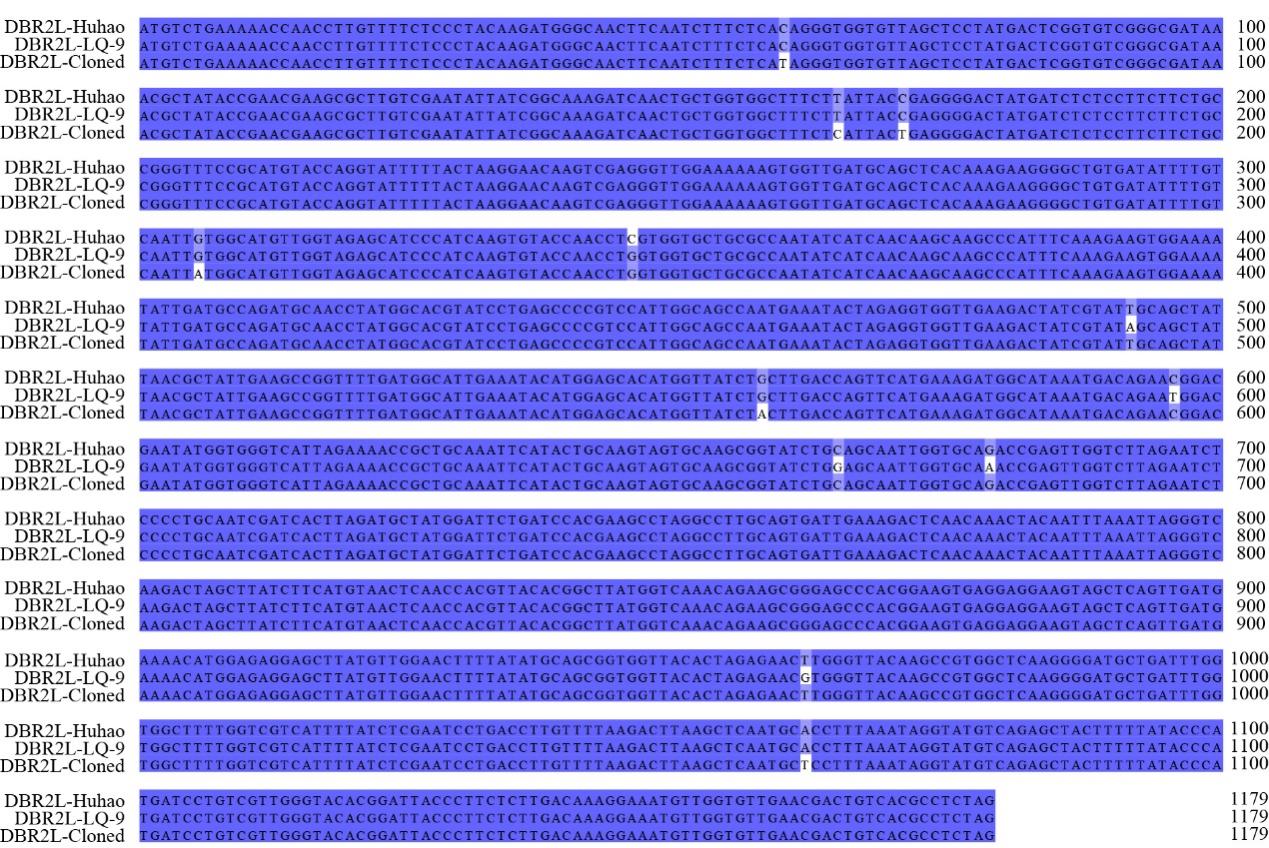


**Supplementary Figure S8 Alignment of reference genome DBR2L CDS sequences with cloned DBR2L sequence.**


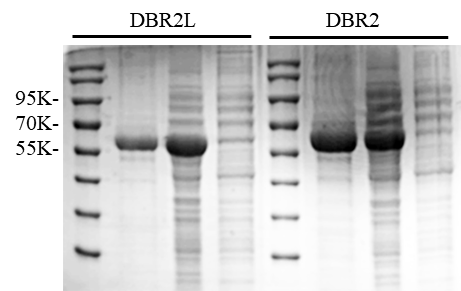


**Supplementary Figure S9 SDS-polyacrylamide gel electrophoresis of DBR2 and DBR2L.** The SDS-10% polyacrylamide gel was stained by Coomassie Blue R-250 after electrophoresis. Each enzyme occupies 4 lanes: marker, purified enzyme, sonicated crude extract and eluent.


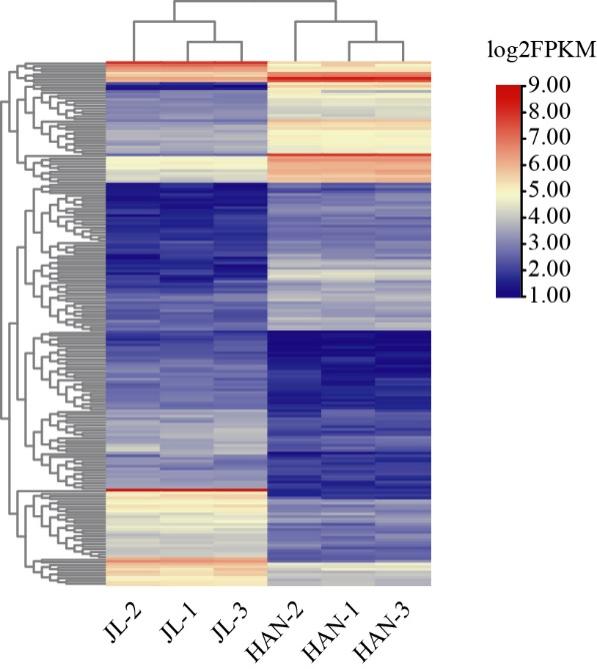


**Supplementary Figure S10 Heatmap of expression level of differently expressed TFs.**

**Supplementary Table S1 Information of collected wild *Artemisia annua***

| Name | Sampling site | Latitude/Longitude | Artemisinin content (%) |
| --- | --- | --- | --- |
| JL | Yongji County, Jilin Province | N43°47′15.14″  E126°0′26.22″ | 0.03±0.03 |
| GS | Dangchang County, Gansu Province | N33°37'39.85″  E104°35'23.76″ | 0.03±0.06 |
| BJ | Beijing | N40°21′19.10″  E116°0′33.40″ | 0.14±0.02 |
| HEN | Tongbo County, Henan Province | N40°21′19.10″  E116°0′33.40″ | 0.26±0.10 |
| HUB | Enshi County, Hubei Province | N30°20′41.17″  E109°26′29.40″ | 0.34±0.10 |
| HAN | Qiongzhong County, Hainan Province | N19°02′29.00″  E109°46′23.16″ | 0.53±0.18 |

**Supplementary Table S2 Basic information of transcriptome data**

| Sample | JL-1 | JL-2 | JL-3 | HAN-1 | HAN-2 | HAN-3 |
| --- | --- | --- | --- | --- | --- | --- |
| Raw Reads Number | 40,664,652 | 36,211,710 | 40,664,510 | 47,194,362 | 40,251,356 | 40,621,718 |
| Raw Bases Number | 6,099,697,800 | 5,431,756,500 | 6,099,676,500 | 7,079,154,300 | 6,037,703,400 | 6,093,257,700 |
| Clean Reads Number | 39,419,996 | 34,574,366 | 39,251,410 | 45,752,120 | 38,908,486 | 39,476,776 |
| Clean Reads Rate(%) | 96.94 | 95.48 | 96.53 | 96.94 | 96.66 | 97.18 |
| Clean Bases Number | 5,912,999,400 | 5,186,154,900 | 5,887,711,500 | 6,862,818,000 | 5,836,272,900 | 5,921,516,400 |
| Low-quality Reads Number | 115,878 | 111,224 | 133,770 | 170,666 | 139,646 | 130,852 |
| Low-quality Reads Rate(%) | 0.28 | 0.31 | 0.33 | 0.36 | 0.35 | 0.32 |
| Ns Reads Number | 124,720 | 277,090 | 144,294 | 147,158 | 131,414 | 114,342 |
| Ns Reads Rate(%) | 0.3 | 0.77 | 0.35 | 0.32 | 0.32 | 0.28 |
| Adapter Polluted Reads Number | 1,004,058 | 1,249,030 | 1,135,036 | 1,124,418 | 1,071,810 | 899,748 |
| Adapter Polluted Reads Rate(%) | 2.47 | 3.45 | 2.79 | 2.38 | 2.66 | 2.21 |
| Raw Q30 Bases Rate(%) | 91.15 | 93.39 | 91.21 | 90.79 | 90.86 | 90.75 |
| Clean Q30 Bases Rate(%) | 91.33 | 93.7 | 91.44 | 91.01 | 91.09 | 90.94 |
| Raw GC percent(%) | 43.6 | 44.32 | 43.77 | 43.56 | 43.47 | 43.52 |
| Clean GC percent(%) | 43.45 | 43.93 | 43.6 | 43.4 | 43.3 | 43.37 |

**Supplementary Table S3 FPKM values of *DBR2* and *DBR2L* in RNA-seq data of JL and HAN**

| Gene | JL-1 | JL-2 | JL-3 | HAN-1 | HAN-2 | HAN-3 |
| --- | --- | --- | --- | --- | --- | --- |
| DBR2-like | 26.87668 | 27.75895 | 27.87892 | 41.07179 | 36.77616 | 38.7328 |
| DBR2 | 0.649761 | 1.32589 | 1.17642 | 0.395004 | 0.531217 | 0.19608 |

**Supplementary Table S4 qRT-PCR Primers of *DBR2* in previous studies**

| Forward | Reverse | Specificity |
| --- | --- | --- |
| CTTGGGTTACAAGCTGTGGCTCAAG | ATATAATCAAAACTAGAGGAGTGACC | Yes |
| GCGGTGGTTACACTAGAGAACTT | ATAATCAAAACTAGAGGAGTGACCC | Yes |
| GAACGGACGAATATGGTGGG | GCAGTATGAATTTGCAGCGGT | No |
| GGCATAAATGACAGAACGGACGAAT | CGATTGCAGGGGAGATTCTAAGACC | No |
| ACTGCTGGTGGCTTTCTTA | ACCCTCGACTTGTTCCTTA | No |
| TCAACTGCTGGTGGCTTTCTT | CAGGGGAGATTCTAAGACCAA | No |

**Supplementary Table S5 Information of molecular and fragment ion**

| Compound | Molecular weight | Molecular ion (+H) | Fragment ion | Collision energy | Fragmentor（V） |
| --- | --- | --- | --- | --- | --- |
|  |  |  |  |  |  |
| Arteannuin B | 248.1 | 249.1 | 189 | 8 | 130 |
|  |  |  | 185 | 7 | 130 |
| Artemisitene | 280.1 | 281.1 | 244.8 | 6 | 60 |
|  |  |  | 217 | 16 | 60 |
| Artemisinin | 282.1 | 283.2 | 246.8 | 6 | 75 |
|  |  |  | 218.8 | 11 | 75 |
| Dihydroartemisinic | 236.2 | 237.1 | 219 | 10 | 80 |
| acid |  |  | 163 | 18 | 80 |
| Artemisinic acid | 234.2 | 235.2 | 199 | 16 | 90 |
|  |  |  | 188 | 13 | 90 |
| Artemisinic aldehyde | 218.2 | 219.2 | 145 | 20 | 100 |
|  |  |  | 105 | 36 | 100 |

**Supplementary Table S6 The linear range of content determination**

| Compound | Lower limit of quantification (µg/mL) | Linear range  (µg/mL) | Linear equation | Regression coefficient (R^2^ ) |
| --- | --- | --- | --- | --- |
|  |  |  |  |  |
| Arteannuin B | 0.005 | 0.005-1 | y=287.27x-393.3 | 1 |
| Artemisitene | 0.01 | 0.01-1 | y=124.46x+5.7869 | 1 |
| Artemisinin | 0.01 | 0.01-1 | y=182.21x-425.35 | 0.9999 |
| Dihydroartemisinic acid | 0.005 | 0.005-0.8 | y=76.806x-904.81 | 0.9996 |
| Artemisinic acid | 0.01 | 0.01-0.8 | y=120.32x-1526.4 | 0.9993 |
| Artemisinic aldehyde | 0.01 | 0.01-0.8 | y=159.88x-1447 | 0.9995 |

**Supplementary Table S7 Primers used in this study**

| Experiment | Primer name | Primer sequence (5’-3’) |
| --- | --- | --- |
| qRT-PCR | Q-ADS-F | ACAACGGGCACTAAAGCAAC |
|  | Q-ADS-R | ATGGCTGAGCTCTTCCTTGT |
|  | Q-CYP-F | CACCCTCCACTACCCTTG |
|  | Q-CYP-R | GACACATCCTTCTCCCAGC |
|  | Q-ALDH1-F | GGACTTGCCTCAGGTGTAT |
|  | Q-ALDH1-R | GTGCCTCTAATCCTTGTTC |
|  | Q-DBR2+DBR2L-F | CAACCTATGGCACGTATCCTGAG |
|  | Q-DBR2+DBR2L-R | CCACCATATTCGTCCGTTCTGTC |
|  | Q-DBR2-F | CTTGGGTTACAAGCTGTGGCTCAAG |
|  | Q-DBR2-R | ATATAATCAAAACTAGAGGAGTGACC |
|  | Q-DBR2L-F | ATTGAAGCCGGTTTTGATGGC |
|  | Q-DBR2L-R | GTTTGACCATAAGCCGTGTAACG |
|  | Aaactin-F | CCAGGCTGTTCAGTCTCTGTAT |
|  | Aaactin-R | CGCTCGGTAAGGATCTTCATCA |
| cloning | DBR2L-F | ATGTCTGAAAAACCAACCTTG |
|  | DBR2L-R | CTAGAGGCGTGACAGTCGTTC |
|  | DBR2Lpro-F | TGAAGGATGACCAAAAGCATAAC |
|  | DBR2Lpro-R | GAGAAAGATTGAAGTTGCCCATC |
| protein expression | DBR2L-PET-F | GCCATGGCTGATATCGGATCCATGTCTGAAAAACCAACCTTGTTTT |
|  | DBR2L-PET-R | TGCGGCCGCAAGCTTGTCGACCTAGAGGCGTGACAGTCGTTCA |
| promoter activity | DBR2Lpro-0800-F | CTATAGGGCGAATTGGGTACCTGAAGGATGACCAAAAGCATAAC |
|  | DBR2Lpro-0800-R1 | ATCGATACCGTCGACCTCGAGTATTGAGTTTGATGTTGATCAG |
|  | DBR2Lpro-0800-R2 | ATCGATACCGTCGACCTCGAGTATTGAATTTGATGTTGATCAG |
|  | HAN-1525-0800-F | CTATAGGGCGAATTGGGTACCTTCATATTTAATGCCTTGTAGG |
|  | HAN-1225-0800-F | CTATAGGGCGAATTGGGTACCTAATGTTCACTTGTTAAAAGC |
|  | HAN-925-0800-F | CTATAGGGCGAATTGGGTACCATTGACACAAGAATCGTAAAAATG |
|  | HAN-625-0800-F | CTATAGGGCGAATTGGGTACCATAAGGGGTGAGCATAACGGAT |
|  | HAN-325-0800-F | CTATAGGGCGAATTGGGTACCCATTTATCTACCTTGTTATTAATG |
